# Disrupting the Undruggable: Emerging Modalities for Targeting Protein–Protein Interactions in Oncology

**DOI:** 10.3390/biology15100759

**Published:** 2026-05-09

**Authors:** Mohamed El-Tanani, Syed Arman Rabbani, Adil Farooq Wali, Yahia El-Tanani, Shrestha Sharma

**Affiliations:** 1 RAK College of Pharmacy, Ras Al Khaimah Medical and Health Sciences University, Ras Al Khaimah P.O. Box 11172, United Arab Emirates; 2 Royal Cornwall Hospital Trust, NHS, Truro TR1 3LJ, UK; 3 Amity Institute of Pharmacy, Amity University, Panchgaon, Gurgaon 122413, India

**Keywords:** protein–protein interactions, oncology, PROTACs, molecular glues, peptidomimetics, AI drug design, Venetoclax, AMG 510, cancer therapeutics, drug resistance, graph neural networks, precision oncology

## Abstract

Protein–protein interactions (PPIs) are critical for cellular signaling, apoptosis regulation, and immune function in the body, and dysregulation is a hallmark of cancer. The large, dynamic, and shallow nature of PPI interfaces rendered them “undruggable” by conventional small molecules in the past. Recent advances in structural biology, chemical innovation, and artificial intelligence have revolutionized the landscape of PPI-directed drug discovery. This review summarizes the mechanistic roles of PPIs in oncogenesis, critically discusses novel therapeutic interventions, such as small molecules, peptidomimetics, stapled peptides, proteolysis-targeting chimeras (PROTACs), molecular glues, and AI-based drug optimization strategies, altering the druggable proteome in oncology. Therapeutics with clinically well-validated action, including Venetoclax and AMG 510, and next-generation candidates demonstrate the translational applications of these approaches. Some of the key challenges, such as interface complexity, specificity, bioavailability, and resistance, are addressed together with countermeasures like rational design, combination therapies, enhanced delivery systems, and biomarker-based patient selection. To this end, the incorporation of multi-omics data and artificial-intelligence (AI)-driven modeling technologies is revolutionizing the personalized cancer therapeutics development space. Collectively, these advances mark a paradigm shift: PPIs, once considered inaccessible, are now at the forefront of precision oncology, offering new hope for patients with previously intractable malignancies.

## Simple Summary

Protein–protein interactions (PPIs) orchestrate how cells sense signals, decide whether to divide or die, and communicate with the immune system. In many cancers, these interactions are rewired by mutations and other changes, creating vulnerabilities that modern therapies can target. PPIs were previously considered difficult to target due to their broad and dynamic interaction interfaces. Over the last ten years, advances in structural biology, chemistry, and artificial intelligence have enabled blocking, degrading, or even stabilizing PPIs using small molecules, engineered peptides, targeted protein degraders (PROTACs), and molecular glues. This review outlines the biology of PPIs in cancer, compares emerging therapeutic modalities, and evaluates real-world successes and limitations. We furthermore touch on practical challenges related to specificity, delivery, and resistance and suggest paths for improvement, including systems-level network mapping, AI-assisted design, and targeted protein stabilization. Collectively, these advancements turn PPIs from difficult targets into actionable nodes for precision oncology.

## 1. Introduction

Protein–protein interactions (PPIs) play a role as a central player in the orchestration of cellular processes, as they form the basis on which signaling networks can function, regulating cell growth, differentiation, apoptosis, and immune responses [1,2]. In cancer biology, such interactions are often dysfunctional and produce aberrant signaling cascades that initiate tumorigenesis and advance tumorigenesis and metastasis. PPIs regulate critical signaling pathways, including the p53 tumor suppressor network, BCL-2 family-mediated apoptosis, and RAS-driven oncogenic signaling. Consequently, they represent an attractive yet historically elusive class of therapeutic targets [3].

Characteristics of protein interfaces have consistently hindered the effort to target PPIs. PPI interfaces (compared to enzyme active sites or receptor pockets) are usually large, flat, and do not have well-defined cavities, causing them to be resistant to traditional small-molecule drug-design approaches [4]. Such surfaces frequently possess several weak interactions in large regions, resulting in the need for inhibitors to have high affinity and specificity without interfering with fundamental physiological functions. Over time, this complexity led to PPIs being regarded as challenging therapeutic targets, which constrained innovation in this area for decades [5,6].

But in the last ten years, a paradigm shift has occurred. In contrast, structural biology advances, including cryo-electron microscopy (cryo-EM) and computational modeling, are yielding unprecedented insights into the dynamic characteristics of PPI interfaces [7]. Meanwhile, advances in chemical biology have resulted in new approaches to modulate PPIs with remarkable precision. They comprise peptidomimetics and stapled peptides that mimic natural protein structures, proteolysis targeting chimeras (PROTACs) that induce targeted protein degradation, and molecular glues that stabilize E3 ligase–target protein interactions [8]. Additionally, the discovery of cryptic pockets and ligand architecture optimization based on AI has not only decreased the time and cost of conventional drug-design pipelines but also accelerated drug design [9,10].

**Figure 1:**
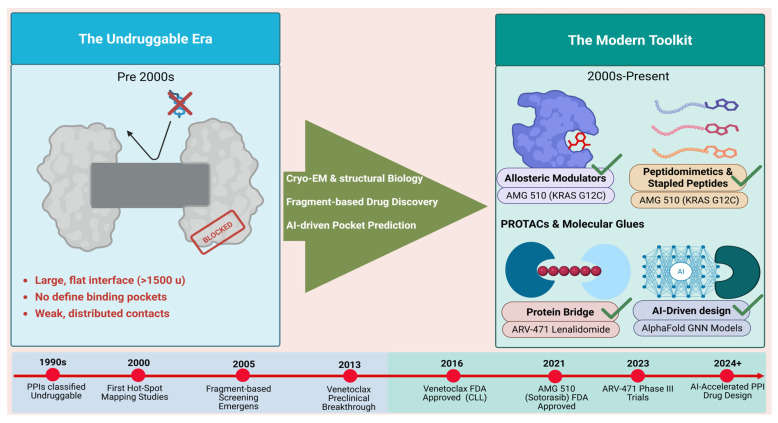
Transition from “undruggable” PPIs with flat interfaces to modern targeting strategies, including allosteric modulators, PROTACs, and AI-driven design.

The clinical success of agents such as Venetoclax, a selective BCL-2 inhibitor, and AMG 510 (Sotorasib), the first KRAS G12C inhibitor, have made PPI targeting in oncology feasible in clinical settings [11]. These drugs have not only validated the concept but also opened new avenues for addressing previously intractable oncogenic drivers. New candidate products like ARV-471, a PROTAC targeting estrogen receptor alpha, and ALRN-6924, a stapled peptide that works by disrupting p53/MDM2 interactions, are also examples of the translational potential of such strategies [11,12]. A schematic timeline illustrating the transition from historically “undruggable” PPIs (characterized by flat, featureless interfaces) to modern targeting strategies such as allosteric modulators, PROTACs, and AI-driven designs is shown in the Figure 1 below.

Nevertheless, several challenges still remain in the way. Problems of interface complexity, off-target effects, poor bioavailability, and resistance mechanisms still remain challenging factors preventing continued improvement [13]. Addressing these problems requires a combination of approaches that are at the interface of rational design, biomarker-based patient selection, and novel delivery systems [14]. Furthermore, the new horizon of PPI-targeted therapy involves utilizing multi-omics data with AI-based predictive models to personalize treatment and predict resistance going forward [15,16].

This review aims to provide a comprehensive overview of the mechanistic roles of PPIs in cancer, emerging therapeutic modalities, and real-world case studies, while critically examining the challenges and strategies for overcoming them. By highlighting the translational relevance and clinical impact of PPI-targeting approaches, we seek to underscore their potential to redefine the therapeutic landscape in oncology.

## 2. Mechanistic Overview of PPIs in Cancer Biology

PPIs can be classified as obligate vs. non-obligate or transient vs. permanent, and these categories have therapeutic implications. It is well established that non-obligate, transient complexes often use short linear motifs (SLiMs) to access shallow and adaptable surfaces, while obligate assemblies typically form stable and buried interfaces [17]. Cancer modifies PPIs by (a) mutating interface hotspots; (b) shifting post-translational modification states to gate docking, such as phosphorylation-dependent binding; and (c) changing protein abundance to reshape local neighborhoods within the interactome. These properties hinder classical active site inhibition but also uncover cryptic allosteric pockets and cooperative nodes that can be utilized by small molecules, peptidomimetics, degraders, and stabilizers [18].

### Experimental Mapping of PPIs: Methods and Use-Cases

Unbiased interactome discovery complements target-driven design. Binary detection techniques (yeast two-hybrid and protein-fragment complementation) reveal direct partnerships; complex-centric approaches (affinity purification–mass spectrometry) and proximity labeling (BioID/TurboID/APEX) uncover neighborhood contexts and condition-specific rewiring. Structural methods (cryo-EM, X-ray, and NMR) and crosslinking mass spectrometry define interface geometry and hydration networks, which guide fragment and covalent warhead placement and the design of degrader ternary complex elements [19]. A summary of key experimental methods used for protein–protein interaction discovery and validation is presented in Table 1.

Protein–protein interactions (PPIs) regulate signaling networks that orchestrate the complex pathways that regulate cellular performance. With respect to cancer, dysregulated PPIs may play a major role in the signaling pathways of uncontrolled proliferation, evasion of apoptosis, sustained angiogenesis, immune escape, and metastasis as hallmark signaling pathways [20]. These relationships frequently include proteins of oncogenic origin, tumor suppressors, and transcription factors, as well as signaling adaptor factors to form dynamic complexes in order to control gene expression and cellular fate [21].

One of the best-known examples is the BCL-2 family of proteins that control mitochondrial-mediated apoptosis. Anti-apoptotic components such as BCL-2 and BCL-XL, meanwhile, bind and sequester pro-apoptotic proteins such as BAX and BAK to avoid cell death [21]. BCL-2 overexpression promotes survival of many hematologic malignancies. Targeting this PPI has given rise to Venetoclax, a selective BCL-2 inhibitor that re-establishes apoptotic signaling [22].

The interplay between mutant KRAS and subsequent effectors such as RAF and PI3K mediates oncogenic signaling in several solid tumors. The KRAS G12C mutation, which is common in non-small cell lung cancer (NSCLC), changes the protein’s shape and influences its interaction with effector proteins. Interfering with the interaction is now a medical focus, and finally, AMG 510 (Sotorasib) binds KRAS G12C not only irreversibly but also suppresses its signaling [23,24].

PPIs are also involved in immune modulation. The interaction between PD-1 on T cells and PD-L1 on tumor cells suppresses immune response, helping tumors escape immune surveillance [25]. Monoclonal antibodies might work to block this interaction, but small molecules and more recent developments have begun to look more carefully into how these interactions can be altered through specialized mechanisms [26].

When it comes to transcriptional regulation, PPIs involving MYC, p53, and NF-κB are critical to oncogenesis. MYC binds to MAX and forms heterodimers to manage gene expression, whereas mutant p53 interacts abnormally with other transcription factors to drive tumorigenesis [27]. Targeting these interactions continues to be challenging because of their complexity and uncharacterized binding pockets [28].

**Figure 2:**
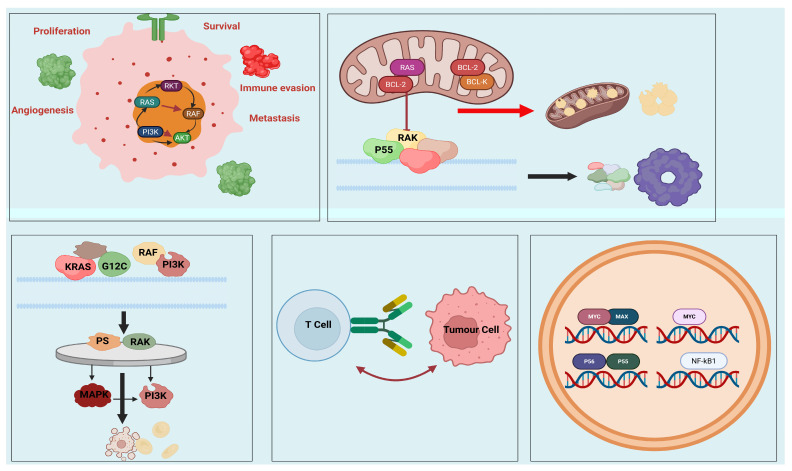
Schematic Representation of Oncogenic RAS Signaling, Effector Interactions, and Therapeutic Targeting Strategies in Cancer.

Overall, PPIs represent a rich landscape of therapeutic targets in cancer biology. It is vital to fully characterize their structure and function for constructing viable therapies that disrupt the oncogenic signaling system and restore normal cellular function (Figure 2).

Unlike prior reviews that primarily focus on individual classes of PPI-targeting agents, this work provides an integrated and cross-modality perspective encompassing small molecules, PROTACs, molecular glues, and peptidomimetics within a unified framework [11]. The novelty of this review lies in its emphasis on comparative evaluation across modalities, incorporation of recent clinical case studies (2020–2025), and detailed analysis of resistance mechanisms and translational limitations. Furthermore, the integration of AI-driven drug design and multi-omics approaches offers a forward-looking perspective that is not comprehensively addressed in existing literature.

## 3. Emerging Modalities for Targeting PPIs

Extracellular and membrane-proximal PPIs including immune checkpoints remain prime territory for antibody engineering. Monoclonals, nanobodies, and bispecifics can block or bridge PPIs with high specificity, enable receptor clustering, or recruit immune effector functions. These biologics complement intracellular modalities by addressing targets inaccessible to small molecules or peptides and by reshaping tumor immune interactions [29]. RNA therapeutics modulate PPIs by altering the abundance of one partner or by expressing decoys. Small interfering RNA (siRNA) and antisense oligonucleotides (ASOs) reduce expression of oncogenic PPI components or adaptors; messenger RNA (mRNA) delivery can express dominant-negative or decoy binders to sequester interfaces. These strategies provide reversible, programmable control and can be paired with targeted delivery systems to tumor tissues [30].

Beyond degradation, small molecules and chimeric modalities can stabilize beneficial PPIs—restoring tumor suppressor complexes or enhancing proteostatic quality control. Approaches include molecular glues that increase partner affinity and deubiquitinase-recruiting chimeras (DUBTACs) that prolong target half-life. Stabilization expands the intervention space from turning off oncogenic PPIs to turning on protective ones [31]. The therapeutic targeting of protein–protein interactions (PPIs) has evolved significantly over the past decade, driven by innovations in chemistry, structural biology, and computational modeling [32]. Below, we explore four major modalities that are redefining the druggable landscape in oncology. A comparative overview of these modalities is provided in Table 2.

### 3.1. Small Molecules

Small molecules remain the cornerstone of modern drug discovery, primarily due to their favorable pharmacokinetic properties, oral bioavailability, and ease of manufacturing. Historically, their application to protein–protein interactions (PPIs) was considered highly challenging because PPI interfaces are typically large, flat, and lack the deep, well-defined pockets that small molecules traditionally exploit. This structural complexity limited early efforts to design effective inhibitors [33].

Recent advances in fragment-based drug discovery, high-throughput screening, and structure-guided optimization have revolutionized this space. These approaches allow researchers to identify small molecules that bind to allosteric sites or transient pockets formed during protein conformational changes, thereby modulating PPIs indirectly or stabilizing inactive states of target proteins [34,35].

**Figure 3:**
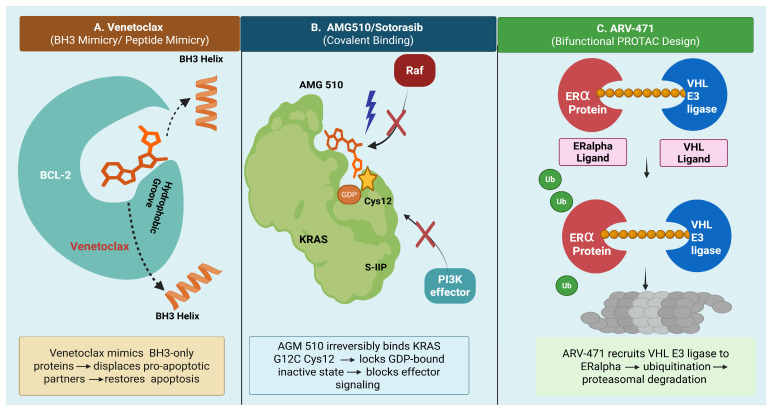
Representative small-molecule strategies for PPI targeting, including Venetoclax (peptide mimicry), AMG 510 (KRAS G12C inhibitor), and ARV-471, illustrating distinct modes of target engagement.

Clinical successes underscore the potential of this modality. Venetoclax, a selective BCL-2 inhibitor, mimics BH3-only proteins to disrupt anti-apoptotic PPIs, restoring apoptotic signaling in hematologic malignancies. Similarly, AMG 510 (Sotorasib) covalently binds to KRAS G12C, locking it in an inactive GDP-bound state and preventing downstream effector interactions—a breakthrough in targeting previously “undruggable” oncogenes [36,37]. Figure 3 illustrates representative small-molecule strategies for PPI targeting.

These examples demonstrate that, with rational design and structural insight, small molecules can effectively modulate PPIs, paving the way for novel therapies that expand the druggable proteome in oncology.

### 3.2. Peptidomimetics and Stapled Peptides

Peptidomimetics refer to synthetic molecules designed to replicate the structural and functional characteristics of natural peptides while mitigating their inherent limitations related to poor stability and rapid degradation. These engineered compounds, therefore, possess enhanced resistance to proteolytic enzymes, provide improved pharmacokinetics, and demonstrate greater specificity for their target protein–protein interactions (PPIs). Peptidomimetics can be used in an effective manner by recognizing critical binding motifs that cannot be found in conventional small molecules or antibodies, thereby disrupting oncogenic PPIs [38].

Stapled peptides are a distinct class of peptidomimetics that include hydrocarbon staples to stabilize α-helical conformations. This structural reinforcement not only maintains the bioactive shape of the peptide and increases cell permeability and protease resistance but also enhances intracellular delivery of the peptide, which is challenging for peptide-based therapeutics [39].

For example, ALRN-6924 is a stapled peptide that disrupts the interaction between p53 and its negative regulators MDM2 and MDMX and thus restores tumor suppressor activity in p53 wild-type cancers. In the same way, SAH-p53-8 mimics the p53 transactivation domain and inhibits MDM2 binding by preventing the binding of MDM2, thereby promoting apoptosis in tumor cells [39].

Not surprisingly, such agents are useful mainly for the inhibition of intracellular PPIs linked to important signaling pathways and can be a potential approach for the treatment of malignancies associated with aberrant protein interactions. Their success highlights the potential of structural innovation in broadening the druggable landscape [37].

### 3.3. PROTACs and Molecular Glues

Proteolysis-targeting chimeras (PROTACs) and molecular glues are novel techniques of protein–protein interaction (PPI) modulation through targeted protein degradation rather than inhibition. PROTACs are bifunctional molecules constituted by two ligands joined by a linker: one ligand binds the target protein and one ligand recruits an E3 ubiquitin ligase [40]. This direct proximity causes the target to be ubiquitinated, making it prone to proteasomal degradation. PROTACs target the whole protein, unlike classical inhibitors, providing consistent therapeutic impact and greatly diminishing the risk of resistance by compensatory signaling pathways [41].

Clinical examples are found in ARV-110, designed to attack the androgen receptor in prostate cancer, and ARV-471, to antagonize estrogen receptor alpha (ERα) in hormone receptor-positive breast cancer. These agents have exhibited strong activity in preclinical and early-phase clinical trials, which further highlights their potential in addressing limitations associated with conventional therapies [42].

Molecular glues, on the other hand, are small molecules that stabilize interactions between E3 ligases and target proteins directly without requiring a linker for their action.

This newer approach has led to the emergence of clinically effective drugs, such as lenalidomide, and second-generation candidates, such as iberdomide, which alter cereblon-mediated degradation of transcription factors in hematologic malignancies [43].

Collectively, PROTACs and molecular glues expand the druggable proteome, enabling precise modulation of oncogenic drivers and providing new therapeutic opportunities for previously intractable cancers [40].

### 3.4. AI-Driven Drug Design

In drug discovery, artificial intelligence (AI) is transforming the landscape, especially targeting protein–protein interactions (PPIs), which are highly complex and dynamic structures. Conventional computational methods have been limited in their ability to identify cryptic binding pockets and capture conformational dynamics. This space has recently been developed with deep learning and generative AI models to facilitate accurate predictions of PPI interfaces, binding affinities, and ligand structures at speed and scale not previously possible [44].

Among recent developments, AlphaFold2 has resulted in near-atomic resolution predictions of protein structures and druggable site identification on PPI interfaces. Advanced generative models (e.g., DiffDock) and reinforcement learning approaches are now being leveraged to develop novel scaffolds tailored for disrupting PPIs. AI-based virtual screening platforms can process billions of compounds in silico, enormously expediting the time and lowering the cost of hit-to-lead optimization [45].

Furthermore, multi-modal AI systems combine structural biology, omics, and real-world clinical outcomes to predict resistance mechanisms and tailor the treatment to individualized therapy. Emerging applications include the de novo design of stapled peptides, PROTAC linker optimization, and the discovery of molecular glues via unsupervised clustering of chemical space [40]. Key AI tasks and tools used in PPI drug discovery are summarized in Table 3.

**Figure 4:**
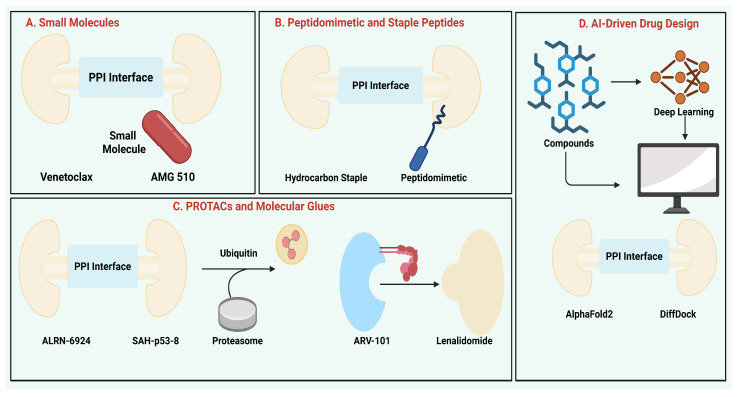
Therapeutic Strategies for Targeting Protein–Protein Interactions: Small Molecules, Peptidomimetics, PROTACs, Molecular Glues, and AI-Driven Drug Design.

With AI-mediated high-throughput experimental validation, the field is advancing toward closed-loop drug discovery, where iterative learning is accelerating innovation at record speeds. These advancements would place AI at the core of growing the druggable PPI domain in oncology (Figure 4).

## 4. Case Studies of PPI-Targeting Drugs in Oncology

Cross-modality comparisons clarify trade-offs. BH3 mimetics target well-defined binding grooves but can cause hematologic toxicity. KRAS G12C inhibitors exploit unique residue chemistry; however, they often face resistance through secondary mutations and receptor tyrosine kinase bypass. ERα degraders achieve strong pathway suppression but are limited by pharmacokinetic challenges. Stapled peptides effectively target intracellular helices, although they frequently suffer from poor oral bioavailability [46]. Incorporating biomarkers, rational combinations, and modality-appropriate delivery mitigates these liabilities. This integrated case-based comparison provides a unique framework to evaluate modality-specific strengths and limitations in real-world clinical contexts [47].

Distinct differences are observed across modalities. Small molecules act through direct inhibition and exhibit favorable pharmacokinetics, with the highest level of clinical maturity. PROTACs enable targeted protein degradation but are limited by their larger size and complex pharmacokinetic profiles. Molecular glues also promote protein degradation and generally possess more drug-like properties, although they are constrained by a narrower target scope and limited clinical validation. Stapled peptides can disrupt or stabilize protein–protein interactions with high specificity but are hindered by poor bioavailability and delivery challenges [43,44].

The clinical validation of protein–protein interaction (PPI)-targeting agents marks a turning point in oncology drug development. Below, we examine five representative case studies that illustrate the diversity of modalities, mechanisms, and therapeutic impact of these agents [20].

### 4.1. Venetoclax (BCL-2 Inhibitor)

Mechanism of Action: Venetoclax is a BH3 mimetic that selectively inhibits the anti-apoptotic protein BCL-2, thereby displacing pro-apoptotic proteins such as BAX and BAK and restoring mitochondrial apoptosis in malignant cells [46,48].

Clinical Impact: Venetoclax is approved for the treatment of chronic lymphocytic leukemia (CLL) and acute myeloid leukemia (AML), particularly in patients with high-risk features such as TP53 mutations or those ineligible for intensive chemotherapy. Clinical trials have demonstrated its ability to achieve deep and durable responses, especially in combination regimens [48,49].

Recent Developments (2020–2025): Recent studies have expanded the use of Venetoclax in AML maintenance settings and in combination with targeted agents such as FLT3 and IDH inhibitors [49,50]. Biomarker-driven approaches, including BCL-2 expression and apoptotic priming, are being explored to optimize patient selection. Additionally, ongoing investigations are evaluating its potential in solid tumors, although efficacy remains limited due to tumor heterogeneity and resistance mechanisms [51]. Despite its clinical success, resistance frequently arises through upregulation of alternative anti-apoptotic proteins such as BCL-XL and MCL-1, limiting long-term efficacy. Additionally, hematologic toxicities and dependence on specific biomarker profiles constrain broader applicability [50,51].

### 4.2. AMG 510 (Sotorasib—KRAS G12C Inhibitor)

Mechanism of Action: KRAS mutations are among the most prevalent oncogenic drivers in solid tumors. AMG 510 covalently binds to the cysteine residue in KRAS G12C, locking it in an inactive GDP-bound state and preventing downstream signaling through RAF-MEK-ERK and PI3K pathways [47,52].

Clinical Impact: Approved for non-small cell lung cancer (NSCLC) with KRAS G12C mutations, AMG 510 represents a landmark achievement in targeting a previously challenging oncogene. Its success has spurred a wave of KRAS-targeted drug development [52].

Recent Developments (2020–2025): Recent developments between 2020 and 2025 have focused on overcoming resistance and improving the effectiveness of KRAS-targeted therapies. Combination trials involving immune checkpoint inhibitors and SHP2 inhibitors are being explored to address adaptive resistance mechanisms [14]. At the same time, significant progress has been made in identifying resistance pathways, including secondary KRAS mutations and bypass signaling through receptor tyrosine kinases (RTKs) [14,25,53]. Additionally, the use of artificial intelligence has enabled the design of next-generation KRAS inhibitors with enhanced potency and the ability to target a broader range of mutations. However, therapeutic responses are often limited by the emergence of secondary KRAS mutations and activation of bypass signaling pathways, particularly via receptor tyrosine kinases. These resistance mechanisms reduce durability of response and necessitate combination strategies [53].

### 4.3. ARV-471 (PROTAC—ERα Degrader)

Mechanism of Action: ARV-471 is a proteolysis-targeting chimera (PROTAC) that recruits estrogen receptor alpha (ERα) to an E3 ubiquitin ligase, inducing its ubiquitination and subsequent proteasomal degradation. This approach offers a more complete and sustained suppression of ER signaling compared to selective estrogen receptor degraders (SERDs) [54].

Clinical Impact: In hormone receptor-positive breast cancer, ARV-471 has demonstrated superior efficacy over fulvestrant, particularly in patients with ESR1 mutations. Its oral bioavailability and favorable safety profile make it an attractive alternative to injectable SERDs [54,55].

Recent Developments (2020–2025): Recent findings highlight encouraging clinical progress, with Phase II trials demonstrating significant improvements in progression-free survival [52]. In parallel, combination strategies involving CDK4/6 inhibitors and modulators of the PI3K pathway are being actively explored to enhance therapeutic outcomes. Additionally, biomarker-driven studies are refining patient selection, particularly by evaluating ERα degradation kinetics to better predict treatment response [54,55]. Nevertheless, PROTAC efficacy is influenced by E3 ligase expression and ternary complex stability, which may vary across tumor types. Furthermore, their large molecular size can lead to suboptimal pharmacokinetics and limited tissue penetration [56].

### 4.4. ALRN-6924 (Stapled Peptide—p53/MDM2/MDMX Disruptor)

Mechanism of Action: ALRN-6924 is a hydrocarbon-stapled peptide that mimics the p53 transactivation domain, disrupting its interaction with negative regulators MDM2 and MDMX. This restores p53 tumor suppressor activity, triggering apoptosis in p53 wild-type cancers [18,57].

Clinical Impact: ALRN-6924 has shown promising activity in hematologic malignancies and solid tumors, particularly liposarcoma and AML. Its ability to reactivate p53 offers a novel therapeutic avenue for tumors retaining wild-type p53 [58].

Recent Developments (2020–2025): Recent developments have focused on improving the effectiveness and scope of these therapies through multiple strategies. Combination trials with DNA-damaging agents and immune checkpoint inhibitors are being actively investigated to enhance therapeutic responses. At the same time, efforts toward pharmacokinetic optimization are aimed at improving drug stability and tissue penetration, thereby increasing overall efficacy. In addition, the exploration of stapled peptide libraries is expanding the potential for targeting a broader range of protein–protein interactions (PPIs) [39,58]. Despite promising activity, clinical translation is hindered by poor oral bioavailability, rapid degradation, and challenges in efficient intracellular delivery. These pharmacokinetic limitations restrict their widespread therapeutic use [59].

### 4.5. Iberdomide (Molecular Glue—CRBN/Ikaros/Aiolos Modulator)

Mechanism of Action: Iberdomide is a next-generation cereblon modulator that acts as a molecular glue, enhancing the interaction between cereblon (CRBN) and transcription factors Ikaros and Aiolos. This leads to their ubiquitination and degradation, exerting potent immunomodulatory and antitumor effects [60].

Clinical Impact: Iberdomide has demonstrated efficacy in relapsed/refractory multiple myeloma and lymphomas, offering improved potency and reduced toxicity compared to earlier immunomodulatory drugs (IMiDs) like lenalidomide [60,61].

**Figure 5:**
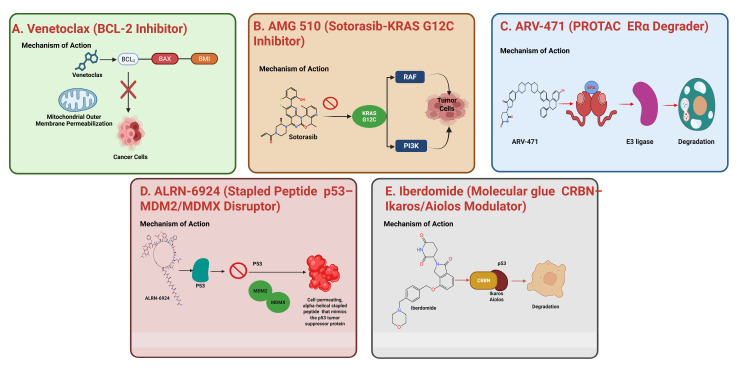
Overview of emerging precision oncology modalities and their mechanisms of action. (**A**) Venetoclax selectively inhibits BCL-2 to restore apoptosis in hematologic malignancies. (**B**) Sotorasib covalently targets KRAS G12C to suppress oncogenic signaling. (**C**) ARV-471 induces estrogen receptor degradation through PROTAC-mediated ubiquitination. (**D**) ALRN-6924 blocks MDM2/MDMX interactions to reactivate p53 signaling. (**E**) Iberdomide, a CELMoD agent, promotes cereblon-mediated degradation of Ikaros and Aiolos in multiple myeloma. Recent advances include FDA approvals, ongoing clinical trials, and expanding therapeutic applications.

Recent Developments (2020–2025): Recent advancements highlight significant progress in this area, with Phase III trials confirming superior clinical outcomes in heavily pretreated patients [18,43,61]. At the same time, AI-driven approaches are enabling the discovery of novel glueable interfaces, thereby expanding the therapeutic scope. In addition, structural studies have provided deeper insights into cereblon–ligand interactions, supporting rational drug design and optimization (Figure 5). However, molecular glues are limited by a narrow substrate spectrum and incomplete understanding of target selectivity. Off-target degradation and resistance due to alterations in cereblon or downstream pathways remain additional concerns [60].

Table 4 provides a comparative overview of different therapeutic modalities, summarizing efficacy, biomarker dependence, and resistance mechanisms across representative case studies. This facilitates cross-modality evaluation by highlighting differences in mechanism of action, clinical performance, and translational limitations. Such comparison enables a more balanced understanding of the strengths and constraints associated with each therapeutic approach.

## 5. Challenges in Targeting PPIs

Therapeutic challenges in targeting protein–protein interactions (PPIs) vary across modalities. Small molecules are constrained by shallow and featureless binding interfaces. PROTACs face limitations related to their large size, complex pharmacokinetics, and dependence on E3 ligases. Molecular glues are restricted by a limited target scope, whereas stapled peptides are hindered by poor bioavailability and delivery challenges [62]. Protein–protein interfaces are often shallow and surrounded by water, which makes it difficult for drugs to bind effectively. Considering factors such as water interactions, electrostatic forces, and protein flexibility can improve drug design, including fragment-based and covalent approaches. For protein degraders, effective ternary complex formation depends on how well the target protein, E3 ligase, and linker fit and cooperate with each other. Understanding these interactions helps in optimizing linker design and improving target- and tissue-specific activity [63]. Although considerable progress has been made in developing PPI-targeted therapeutics, key scientific, pharmacological, and translational challenges persist. These challenges arise from the intrinsic complexity of PPI interfaces, drug delivery constraints, and emerging resistance mechanisms that need to be overcome to realize the full potential for PPI-targeted therapies in oncology [63].

### 5.1. Structural Complexity of PPI Interfaces

Unlike enzyme active sites and receptor pockets, PPI interfaces are typically large, flat, and featureless, spanning 1500–3000 Å^2^ of surface area. However, such regions do not have deep hydrophobic cavities and therefore may prevent small molecules from high-affinity binding. In addition, PPI interfaces themselves are transient and conformationally dynamic, which makes obtaining stabilized binding sites hard. Even with the state-of-the-art structural biology toolkit like cryo-EM and computational modeling, building molecules that interfere directly with these interactions to disrupt them is a formidable challenge [64].

### 5.2. Specificity and Off-Target Effects

Selectivity is important, as many protein domains involved in PPIs are common to multiple signaling pathways. For example, BH3 mimetics designed to target BCL-2 can also affect BCL-XL, which may cause thrombocytopenia. Similarly, KRAS inhibitors may impact other RAS proteins. Such off-target effects can lead to toxicity and limit the safe dose, especially for intracellular targets that antibodies cannot reach [65].

### 5.3. Bioavailability and Pharmacokinetics

Although peptidomimetics and stapled peptides have been developed with better PPI disruption mechanisms, they suffer from poor oral bioavailability and rapid proteolytic degradation. PROTACs, though extremely effective, are large molecules with complex physicochemical properties that restrict tissue penetration and might necessitate intravenous administration. Despite their effectiveness, adequate systemic exposure and intracellular delivery remain major barriers to clinical translation [66].

### 5.4. Resistance Mechanisms

As with other targeted therapies, compounds used to decrease protein–protein interactions (PPIs) are vulnerable to resistance from different routes of action. These comprise mutations within the target protein that alter the binding interface; for example, secondary KRAS mutations can reduce the efficacy of AMG 510. In addition, cancer cells may overexpress compensatory pathways, including the increased expression of other anti-apoptotic proteins, as observed in acute myeloid leukemia (AML) treated with Venetoclax. Alterations in the expression of E3 ligases in tumors treated with PROTACs can further attenuate protein degradation efficiency. These adaptive strategies underscore the need for combination approaches and predictive biomarkers to better anticipate and address resistance to PPI-targeted therapies [67].

### 5.5. Manufacturing and Scalability

**Figure 6:**
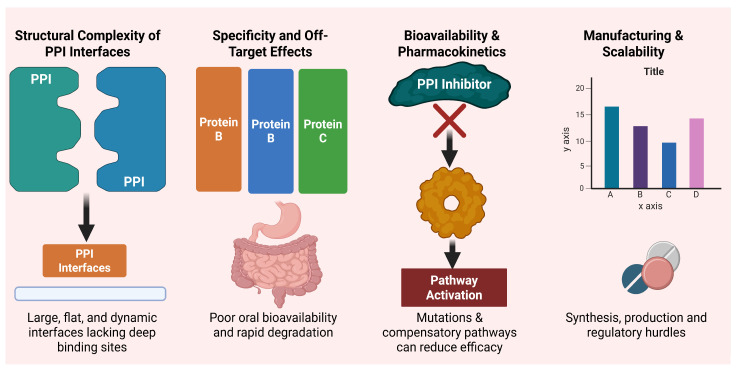
Key challenges in targeting protein–protein interactions (PPIs), encompassing structural complexity, poor pharmacokinetics, biological resistance, and the logistical hurdles of manufacturing and regulation.

Complex modalities including stapled peptides and PROTACs require complex synthesis and purification steps. Scaling up production of these agents without loss in quality of production and cost is necessary for clinical application. Additionally, evolving regulatory frameworks for these emerging modalities introduce further complexity to their commercialization (Figure 6) [14].

### 5.6. Cross-Modality Comparison and Clinical Limitations

Various therapeutic approaches aimed at targeting protein–protein interactions (PPIs) possess apparent advantages and limitations which could affect their clinical translation. Small molecules still remain the foremost modality with respect to oral bioavailability and manufacturability; however, their effectiveness is often limited by the shallow and dynamic nature of PPI interfaces, resulting in suboptimal binding and off-target effects [66]. In comparison, PROTACs promote catalytic degradation of target proteins and can accomplish more sustained pathway suppression, but due to their large molecular size, complex pharmacokinetics, and dependency on E3 ligase expression present crucial challenges for tissue penetration and clinical consistency. Molecular glues offer a more compact alternative for targeted protein degradation with improved drug-like properties, yet their applicability is currently limited by a narrow range of “glueable” targets and incomplete understanding of induced protein–protein interactions. Stapled peptides and other peptidomimetics provide high specificity and are particularly effective in disrupting helical interfaces; however, they suffer from poor oral bioavailability, rapid proteolytic degradation, and delivery barriers [66].

Notably, setbacks observed across these modalities reveal shared challenges in clinical translation, including resistance driven by secondary mutations, activation of compensatory pathways, and changes in protein expression or degradation systems. Moreover, safety concerns arising from both on-target and off-target effects, together with difficulties in large-scale manufacturing and regulatory approval, further hinder development. Taken together, these findings indicate that no single modality is universally ideal, and progress in PPI-targeted therapies will rely on informed modality selection, combination approaches, and ongoing innovations in drug design and delivery [67,68].

## 6. Strategies to Overcome These Challenges

Tumorigenesis requires interactome rewiring across scales. Network principles such as hubs, bottlenecks, and controllability highlight why disrupting a few high-betweenness nodes can collapse oncogenic signaling while sparing normal physiology [69]. Multi-omics integration (genomics, transcriptomics, proteomics, phosphor-proteomics, and spatial proteomics) maps druggable PPI clusters, uncovers pathway bypass routes driving resistance, and informs biomarker-driven patient selection [70].

Given the complexity of targeting protein–protein interactions (PPIs) in oncology, structural, pharmacological and translational barriers continue to be tackled. The following are key approaches that have emerged to address these challenges and drive clinical success [71].

### 6.1. Rational Design and Structure-Guided Optimization

Recent progress in structural biology, for example, cryo-electron microscopy (cryo-EM) and X-ray crystallography, and artificial intelligence (AI)-based modelling (for example, AlphaFold), enables high-resolution visualization of PPI interfaces [72]. These observations allow investigators to visualize elusive, cryptic microstructure features and allosteric sites, which can, in turn, be used for drug targeting. Fragment-based drug discovery and molecular dynamics studies enhance ligand design, leading to increased specificity and affinity. For example, KRAS G12C inhibitors such as AMG 510 were developed using covalent binding strategies informed by structural insights, transforming a previously challenging target into a clinically validated success [73].

### 6.2. Combination Therapies

The efficacy of targeting PPI can be increased and resistance can be reduced by combining PPI-targeting agents with other drugs. For example, Venetoclax is often combined with hypomethylating agents or FLT3 inhibitors in AML to target adaptive mechanisms of survival [74]. PROTACs like ARV-471 are in the process of being tested as concomitant therapies with CDK4/6 inhibitors in ER-positive breast cancer. These adjunct therapies not only increase response but also reduce the possibility of resistance emergence [75].

### 6.3. Biomarker-Guided Patient Selection

Precision oncology depends on the use of predictive biomarkers to select therapy with precision. In the case of PPI-therapy drugs, biomarkers such as BCL-2 expression (for Venetoclax), KRAS G12C mutation status (for AMG 510), and ESR1 mutations (for ARV-471) play a crucial role in identifying patients most likely to benefit from treatment [76]. The integration of multi-omics data—including genomics, transcriptomics, and proteomics—supports real-time assessment of biomarker profiles, enabling adaptive treatment strategies and more personalized patient care [77].

### 6.4. Advanced Drug Delivery Systems

Novel drug delivery approaches are being explored to address bioavailability challenges. These include nanoparticles and liposomes for encapsulation of peptidomimetics and PROTACs, as well as prodrug approaches, which are aimed at increasing oral absorption and metabolic stability [78,79]. Cell-penetrating peptides and cell-penetrating peptide conjugates are being investigated for improved intracellular delivery. This will ensure sufficient systemic availability, reduce degradation and allow for localized delivery to tumor tissue and reduce off-target toxicity [80,81].

### 6.5. AI-Driven Predictive Modeling and Optimization

AI is transforming drug discovery by predicting binding affinity, mimicking molecular interactions, and optimizing pharmacokinetics. Generative AI provides novel scaffolds for PPI disruption, and machine learning algorithms are expected to predict resistance mutations and suggest combination strategies. AI synthesis planning further streamlines manufacturing, allowing cost reduction and acceleration of time-to-market [81].

### 6.6. Regulatory and Manufacturing Innovations

**Figure 7:**
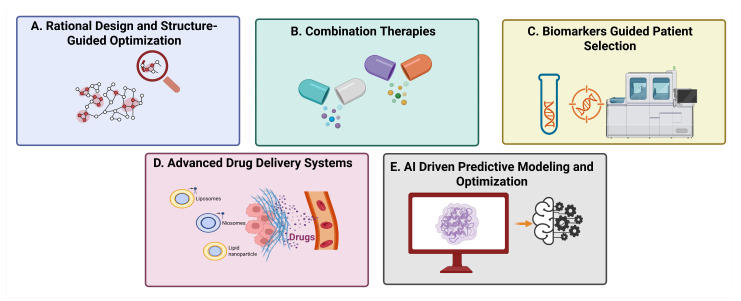
Strategic approaches for enhancing PPI-targeted drug discovery and therapeutic efficacy. (**A**) Advances in structural biology, including cryo-electron microscopy (cryo-EM), X-ray crystallography, and AI-driven modeling, enable identification of druggable pockets and allosteric sites. (**B**) Combination strategies involving PPI-targeting agents improve therapeutic efficacy and help overcome resistance. (**C**) Biomarker-based approaches facilitate identification of patient populations most likely to respond to therapy. (**D**) Nanoparticles, liposomal formulations, prodrug systems, and cell-penetrating technologies enhance drug delivery efficiency and bioavailability. (**E**) Artificial intelligence supports prediction of molecular affinities, simulation of intermolecular interactions, and optimization of synthesis routes.

Modular chemistry approaches, along with automated synthesis platforms, are paving the way for scaling up to complex modalities, such as stapled peptides and PROTACs. At the same time, regulatory agencies are constructing evaluation frameworks for novel mechanisms of action, aiming to ensure safety and efficacy while accelerating approval processes (Figure 7) [82].

These strategies are the basis of rational design, biomarker integration, delivery optimization with advanced algorithms, sophisticated AI optimization, and regulatory innovation, translating a project of PPI-targeted therapy from a conceptual challenge to a clinical reality.

## 7. Future Directions and Clinical Translation

The therapeutic targeting of protein–protein interactions (PPIs) in oncology is entering a transformative era. PPIs, which used to be thought “undruggable,” are at the cutting edge of precision medicine, thanks to progress in structural biology, chemical innovation, and computational modeling. As the field further emerges, several factors will shape its future direction [83,84].

### 7.1. Expansion of the Druggable PPI Landscape

Advances in high-resolution structural biology, including cryo-electron microscopy (cryo-EM) and AI-driven modeling tools such as AlphaFold2, are uncovering previously inaccessible PPI interfaces [85]. These approaches facilitate the detection of cryptic binding pockets and transient conformations that can be leveraged for drug design. Looking ahead, efforts are likely to emphasize system-level mapping of interaction networks to identify key “hub” PPIs that regulate multiple oncogenic pathways. Such strategies will expand the repertoire of druggable targets beyond well-established interactions like BCL-2 and KRAS [86].

### 7.2. Personalized Medicine and Biomarker Integration

Precision oncology will increasingly rely on biomarker-driven patient stratification to optimize the use of PPI-targeted therapies [87]. Predictive biomarkers such as KRAS G12C mutation status, BCL-2 expression, and ESR1 mutations are already being used to guide clinical decision-making for therapies like AMG 510, Venetoclax, and ARV-471. Future strategies will incorporate multi-omics approaches—including genomics, transcriptomics, and proteomics—to refine patient stratification and enable dynamic monitoring of PPI networks during treatment. In addition, real-time biomarker assessment through liquid biopsy will support adaptive therapeutic adjustments, ultimately improving outcomes and minimizing resistance [88,89].

### 7.3. AI-Enhanced Drug Discovery and Predictive Modeling

Artificial intelligence (AI) will play a pivotal role in accelerating PPI drug discovery. Generative AI models and reinforcement-learning algorithms are already applied to novel scaffolds for PPI disruption, optimal PROTAC linker optimization, and molecular glue candidate prediction [90]. Emerging platforms enable integrated frameworks that combine structural data, chemical space exploration, and clinical outcomes to establish closed-loop systems for continuous drug optimization [91]. AI-powered predictive modeling will also predict mechanisms of resistance and be able to be used for rational combination and next-generation inhibitor design [91].

### 7.4. Novel Modalities and Delivery Platforms

From a broader perspective, future modalities may include RNA-based therapeutics, bispecific molecules, as well as nanotechnology-enabled delivery for small molecules and peptides. These modifications focus on overcoming bioavailability issues and enhancing delivery of large molecules such as stapled peptides and PROTACs within the cell [92]. The introduction of smart nanoparticles and targeted conjugates will enable tumor-specific delivery, reducing systemic toxicity. Moreover, using theranostic platforms that combine imaging and therapy in a therapeutic approach may potentially allow real-time monitoring of drug distribution and effectiveness [93].

### 7.5. Combination Therapies and Synthetic Lethality

Combination strategies will be common practice to counteract resistance. Combining PPI-targeted agents with immune checkpoint inhibitors, kinase inhibitors, or DNA-damaging agents can also produce synergistic effects [94]. Another promising strategy involves exploiting synthetic lethality—the simultaneous disruption of two complementary pathways leading to cell death. For instance, combining Venetoclax with FLT3 inhibitors in acute myeloid leukemia (AML), or pairing PROTACs with CDK4/6 inhibitors in breast cancer, exemplifies this approach [95].

### 7.6. Regulatory and Commercial Considerations

As the development of PPI-targeting drugs expands, regulatory frameworks must evolve to address novel mechanisms of action and increasingly complex therapeutic modalities [96]. Clear guidance on pharmacodynamic characterization, biomarker validation, and manufacturing scale-up will be essential. Ultimately, commercial success will hinge on cost-effective synthesis, robust supply chains, and equitable access, particularly in resource-limited settings [97].

Integration of structural insights, computational power, biomarker-driven precision, and novel delivery systems lays the groundwork for the future of PPI-targeted oncology. With these developments in mind, the oncology community has the potential to see PPI modulation from a niche mode to transform it into a standard aspect of cancer therapy, providing new hope for patients with ever-more intractable tumors [98].

## 8. Conclusions

This review uniquely bridges mechanistic insights, clinical evidence, and emerging technologies to provide a comprehensive and comparative understanding of PPI-targeting strategies in oncology. Protein–protein interactions (PPIs) have emerged as a new and promising area in cancer therapeutics. Once considered difficult to target, PPIs are now actively pursued using a range of innovative modalities, including small molecules, peptidomimetics, stapled peptides, PROTACs, molecular glues, and next-generation AI-driven drug design approaches [99]. These approaches can effectively modulate key oncogenic pathways, restore tumor suppressor functions, and overcome resistance mechanisms, thereby expanding the therapeutic options available to oncologists. The strong clinical performance of agents such as Venetoclax, AMG 510, and ARV-471 underscores the importance of rational drug design, biomarker-driven patient selection, and combination strategies in achieving meaningful clinical responses [100]. Although complex interaction with biology and bioavailability remain major challenges, new technologies and integrative concepts are paving the way for more specific and personalized cancer therapies. Furthermore, the combination of structural biology, systems medicine and artificial intelligence is likely to expedite the emergence and optimization of PPI targeting agents [101]. As the field advances, clear regulatory guidance, scalable manufacturing, and equitable access will be essential to translate these innovations into meaningful clinical outcomes. Once considered challenging to target, PPIs are now being successfully modulated, and continued innovation alongside cross-disciplinary collaboration will enable the oncology community to fully leverage PPI targeting for improved cancer therapy [101,102]. This will not only transform cancer care services but will also improve patient outcomes. No single therapeutic modality is universally optimal for targeting protein–protein interactions, as each approach presents distinct advantages and limitations. Future success will rely on rational modality selection, biomarker-guided strategies, and combination approaches to overcome resistance and enhance clinical outcomes [102]. By integrating cross-modality comparisons with clinical and technological perspectives, this work offers a distinct contribution beyond existing narrative reviews.

## Figures and Tables

**Table 1:** Experimental Methods for PPI Discovery and Validation.

Assay	Detects	Throughput	Strengths	Limitations	Best-Fit Use-Cases	Key References
Yeast two-hybrid	Binary direct PPIs	High	Simple, scalable	False positives; nuclear context	Partner discovery	[3]
AP-MS	Complex-centric associations	Medium	Native; network-level insight	Indirect interactions; affinity bias	Interactome mapping	[3]
Proximity labeling (BioID/TurboID/APEX)	Spatial proximity	Medium–High	Captures weak/transient interactions	Labeling radius; background noise	Dynamic networks; organelles	[20]
Crosslinking MS	Distance-constrained contacts	Medium	Provides interface geometry	Crosslink bias; technical complexity	Structural restraints	[19]
Cryo-EM/X-ray/NMR	Atomic-level structures	Low–Medium	High-resolution structural detail	Sample preparation challenges	Structure-guided drug design	[19]

**Table 2:** Therapeutic Modalities for PPIs: Comparative Overview.

Modality	PPI Effect	Intracellular Access	Oral Feasibility	Safety Themes	Examples	Stage	Key References
Small molecules	Block/allosteric modulation	High	Often yes	Off-target effects; metabolism issues	BH3 mimetics; KRAS G12C inhibitors	Approved	[5]
Stapled peptides	Block (α-helix mimic)	Moderate–High (optimized)	Rare	Immunogenicity; poor PK	ALRN-6924	Phase I/II	[20]
PROTACs	Targeted protein degradation	High	Rare	Ligase-related toxicity; on-target effects	ARV-471	Phase II	[11,14]
Molecular glues	Stabilize ligase–substrate interaction	High	Sometimes	Neosubstrate specificity risks	Iberdomide	Phase III/Approved	[10,33]
Antibodies/nanobodies	Block/bridge/ADCC	Extracellular	Not applicable	Infusion reactions; immune effects	PD-(L)1 inhibitors; bispecific antibodies	Approved	[7]
RNA therapeutics	Knockdown/decoy	High (with delivery systems)	Parenteral	Immune activation; delivery challenges	siRNA, ASO, mRNA	Clinical/Preclinical	[30]
Stabilizers (DUBTACs)	Stabilize protective PPIs	High (emerging)	Unknown	Risk of over-stabilization	Experimental DUBTACs	Preclinical	[31]

**Table 3:** AI Tasks and Tools for PPI Drug Discovery.

Task	Model Class	Representative Tools	Deliverable	Validation	Pros/Cons	Key References
Complex structure prediction	Deep learning (multimer models)	AlphaFold-Multimer; ML-assisted docking	PPI interface structures and binding hypotheses	Benchmarking vs. experimental structures; limited for transient PPIs	Fast and scalable; may miss conformational dynamics	[45]
Druggability scoring	Graph neural networks (GNNs); ensemble ML	Graph-based pocket predictors; hotspot mapping tools	Identification of druggable sites and cryptic pockets	Emerging validation datasets	Interpretable features; limited generalization	[15,16,21]
Ligand/design generation	Diffusion models; reinforcement learning	DiffDock; generative RL frameworks	Novel small molecules, peptides, and modulators	Increasing prospective validation studies	Expands chemical space; requires filtering/optimization	[44]
Degrader design (PROTACs/glues)	Physics–ML hybrid models	Ternary complex modeling tools	Linker optimization; cooperativity prediction	Preclinical and early experimental validation	Sensitive to geometry and ligase selection	[11,41,43]
ADMET/Toxicity prediction	Multitask machine learning	Property prediction platforms	PK/PD properties; toxicity flags	Widely used in industry pipelines	Fast screening; risk of bias and uncertainty	[13,24]

**Table 4:** Case-Study Matrix: Efficacy, Biomarkers, and Resistance.

Target/Pathway	Agent	Modality	Cancer Type	Biomarker	Efficacy	Resistance	Notes	Key References
BCL-2	Venetoclax	Small molecule	CLL/AML	BCL-2 expression; TP53 status	Deep remissions in subsets	BCL-XL/MCL-1 bypass	Combination with HMAs, FLT3 inhibitors	[48,49,51]
KRAS G12C	Sotorasib	Covalent small molecule	NSCLC	KRAS G12C mutation	Responses in pretreated patients	Secondary KRAS mutations; RTK/SHP2 bypass	Ongoing combination studies	[47,52,53]
ERα	ARV-471	PROTAC	HR+ Breast cancer	ESR1 mutation/degradation	Enhanced ER degradation and response	Ligase dependency; ternary complex stability	Combined with CDK4/6 inhibitors	[54,55]
p53–MDM2/X	ALRN-6924	Stapled peptide	WT p53 tumors	Wild-type TP53	Reactivation of p53 pathway	Delivery limitations; PK challenges	Combined with DNA-damaging agents	[58,59]
CRBN–Ikaros/Aiolos	Iberdomide	Molecular glue	Multiple myeloma/lymphoma	CRBN expression	Activity in refractory disease	Limited neosubstrate scope	Improved potency vs. earlier IMiDs	[60,61]

## Data Availability

No new data were created or analyzed in this study.

## Abbreviations

The following abbreviations are used in this manuscript:

ADCC	Glycine-to-Cysteine substitution at codon 12
AML	Acute Myeloid Leukemia
AP-MS	Affinity Purification–Mass Spectrometry
APEX	Engineered Ascorbate Peroxidase
ASO	Antisense Oligonucleotide
AI	Artificial Intelligence
AR	Androgen Receptor
BAK	BCL-2 Homologous Antagonist/Killer
BAX	BCL-2-Associated X Protein
BCL-2	B-cell Lymphoma 2
BCL-XL	B-cell Lymphoma-extra Large
BioID	Proximity-dependent Biotin Identification
BH3	BCL-2 Homology Domain 3
CLL	Chronic Lymphocytic Leukemia
cryo-EM	Cryo-Electron Microscopy
CRBN	Cereblon
CDK4/6	Cyclin-Dependent Kinases 4 and 6
DUBTAC	Deubiquitinase-Targeting Chimera
DNA	Deoxyribonucleic Acid
Erα	Estrogen Receptor Alpha
E3	Ubiquitin Ligase Enzyme
ESR1	Estrogen Receptor 1 Gene
FDA	Food and Drug Administration
FLT3	Fms-like Tyrosine Kinase 3
GDP	Guanosine Diphosphate
HMA	Hypomethylating Agent
HR+	Hormone Receptor Positive
IMiDs	Immunomodulatory Drugs
KRASG12C	Kirsten Rat Sarcoma Viral Oncogene Homolog Glycine-to-Cysteine substitution at codon 12
MAX	MYC-Associated Factor X
MDM2	Mouse Double Minute 2 Homolog
MDMX	Mouse Double Minute X
mRNA	Messenger RNA
NF-κB	Nuclear Factor Kappa-light-chain-enhancer of Activated B Cells
NMR	Nuclear Magnetic Resonance
NSCLC	Non-Small Cell Lung Cancer
PK	Pharmacokinetics
PD	Pharmacodynamics
PD-1	Programmed Cell Death Protein 1
PD-L1	Programmed Death-Ligand 1
PI3K	Phosphoinositide 3-Kinase
PPI	Protein–Protein Interaction
PROTAC	Proteolysis-Targeting Chimera
RAF	Rapidly Accelerated Fibrosarcoma Kinase
RAS	Rat Sarcoma
RTK	Receptor Tyrosine Kinase
RNA	Ribonucleic Acid
SERD	Selective Estrogen Receptor Degrader
siRNA	Small Interfering RNA
SLiM	Short Linear Motif
SHP2	Src Homology Region 2-containing Protein Tyrosine Phosphatase-2
TP53	Tumor Protein p53 Gene
TurboID	Engineered Biotin Ligase Variant
UPS	Ubiquitin–Proteasome System
X-ray	X-ray Crystallography
